# Dramatic neurological and biological effects by botulinum neurotoxin type A on SH-SY5Y neuroblastoma cells, beyond the blockade of neurotransmitter release

**DOI:** 10.1186/s40360-020-00443-0

**Published:** 2020-09-05

**Authors:** Lei Wang, Carol S. Ringelberg, Bal R. Singh

**Affiliations:** 1 Prime Bio, Inc., North Dartmouth, MA 02747 USA; 2 Genomics and Molecular Biology Shared Resource, Geisel School of Medicine at Dartmouth, Hanover, NH 03755 USA; 3 Institute of Advanced Sciences, Botulinum Research Center, North Dartmouth, MA 02747 USA

**Keywords:** Gene expression profile, *Clostridium botulinum*, Botulinum neurotoxin type A, Host response, Microarray analysis, Neuroblastoma SH-SY5Y cells, Zinc, Neurological signaling pathways, Biological signaling

## Abstract

**Background:**

Gene expression profile analysis on mammalian cell lines and animal models after exposure to botulinum neurotoxin (BoNT) has been investigated in several studies in recent years. Microarray analysis provides a powerful tool for identifying critical signaling pathways involved in the biological and inflammatory responses to BoNT and helps determine the mechanism of the function of botulinum toxins.

One of the pivotal clinical characteristics of BoNT is its prolonged on-site effects. The role of BoNT on the blockage of neurotransmitter acetylcholine release in the neuromuscular junction has been well established. However, the effects of the treatment time of BoNT on the human cellular model and its potential mechanism remain to be defined.

**Methods:**

This study aimed to use gene microarray technology to compare the two physiological critical time points of BoNT type A (BoNT/A) treatment of human neuroblastoma cells and to advance our understanding of the profound biological influences that toxin molecules play in the neuronal cellular system. SH-SY5Y neuroblastoma cells were treated with BoNT/A for 4 and 48 h, which represent the time needed for the entrance of toxin into the cells and the time necessary for the initial appearance of the on-site effects after BoNT application, respectively.

**Results:**

A comparison of the two time points identified 122 functional groups that are significantly changed. The top five groups are alternative splicing, phosphoprotein, nucleus, cytoplasm, and acetylation. Furthermore, after 48 h, there were 744 genes significantly up-regulated, and 624 genes significantly down-regulated (p‹ 0.01). These genes fell into the following neurological and biological annotation groups: Nervous system development, proteinaceous extracellular matrix, signaling pathways regulating pluripotency of stem cells, cellular function and signal transduction, and apoptosis.

We have also noticed that the up-regulated groups contained neuronal cell development, nervous system development, and metabolic processes. In contrast, the down-regulated groups contained many chromosomes and cell cycle categories.

**Conclusions:**

The effects of BoNT/A on neuronal cells extend beyond blocking the neurotransmitter release, and that BoNT/A is a multifunctional molecule that can evoke profound cellular responses which warrant a more in-depth understanding of the mechanism of the toxin’s effects after administration.

## Background

Besides being known as the most potent poison known to humankind which causes botulism, Botulinum neurotoxin (BoNT) is also a multifunctional molecule which can be used to treat medical conditions such as muscle hyperactivity [8], neuromuscular disorders [11], various types of pain [46], and treatment of wrinkles [22]. New applications of BoNT have been continuously discovered in the past decade [17, 47, 54]. The mechanisms for BoNT to cause botulism have been well established. BoNT acts preferentially on peripheral cholinergic nerve terminals to inhibit acetylcholine release resulting in flaccid muscle paralysis [49]. However, the mechanisms of BoNT as an effective treatment for multiple medical conditions are currently unclear.

Among all the seven toxin serotypes produced by *C. botulinum*, both BoNT type A (BoNT/A) and type B (BoNT/B) are used in treating medical conditions, and the duration of BoNT/A has the most sustained action in both laboratory animals and human beings. In an effort to investigate the mechanisms of BoNT as an effective neuromedicine, an increasing number of studies indicate that BoNT is not only a toxin but also a multifunctional molecule that participates in the regulation of gene expressions and metabolic pathways (Y. J [26, 37].; T. H [43].). BoNT/A has been shown to influence cellular dynamics via the modification of cellular apoptosis and cellular proliferation, which can play a role in the expression of genes relevant to abnormal fibroblast proliferation [56]. In the murine alveolar macrophage RAW264.7 cell line, microarray analysis reveals that BoNT/A induces host immune cell response through a TLR2-dependent manner (Y. J [26].). In another study, BoNT/B has been shown to induce the expression of multiple proinflammatory cytokine genes in a murine dry eye model (C. Y [42].). A previous microarray study in SH-SY5Y cells from our group found that there were significant changes in genes involved in neuroinflammatory, ubiquitin-proteasome degradation, and calcium signaling after BoNT/A treatment compared to the untreated control culture [51]. However, most of the current cellular gene expression studies were conducted at one fixed time point; the genomic responses to BoNTs across different time periods have not been fully clarified.

A transcriptomic profiling study that examined the effects of BoNT/A on rat tibialis anterior muscles over 1 year identified dramatic transcriptional adaptation effects to BoNT/A [36]. Another microarray analysis study has investigated the muscle recovery from paresis and atrophy after intramuscular injection of BoNT/A in Juvenile rats. The microarray transcriptional profiling and real-time-PCR analysis identified a sequence of cellular events that leads to the neuromuscular junction regeneration and skeletal muscle functional recovery, and the insulin-like growth factor-1 (IGF-1) signaling pathway is proven to play a central role in the process [50]. However, none of the studies have examined the progression of the BoNT effect on gene expression with time. Microarray analysis that focuses on the cellular response over a period of time will provide new insight into the critical signaling pathways involved in the biological and inflammatory responses to BoNT.

In vitro models for the entry of BoNT into neurons have shown that at least 4-h is needed for BoNT to enter the neuron cells [14]. Our group has also observed the binding and co-localization of BoNT/A with intracellular structures in SH-SY5Y cells after 4-h of treatment [52]. In both the clinical administrations of BoNT and the in vivo or in vitro BoNT treatment systems, the on-site effect usually takes about 24 to 72 h [39]. In addition, a previous study from our group has observed a significant increase of proinflammatory cytokine release from the SH-SY5Y cells after 48-h of BoNT/A treatment [52] indicating the effectiveness of the duration on evoking the cellular response.

Our current study aims to explore the differences of gene expression profiles in human neuroblastoma SH-SY5Y cells after exposure to BoNT/A at two physiological critical time points: at 4-h when BoNT/A has made its entry into the cells and at the time of the observation of evoked host responses by BoNT/A which is usually 48-h. Comparative analysis of gene expression at the two time points identified 122 functional groups that are significantly changed in the neuronal cells. The top five groups are alternative splicing, phosphoprotein, nucleus, cytoplasm, and acetylation. Furthermore, 744 genes were significantly up-regulated after 48 h, and 624 genes were significantly down-regulated (p‹ 0.01). These genes generally belonged to nervous system development, proteinaceous extracellular matrix, signaling pathways regulating pluripotency of stem cells, cellular function and signal transduction, and apoptosis, suggesting a substantial biological and neurological impact of BoNT treatment.

## Methods

### Materials

The 150 kDa BoNT/A holotoxin was purchased from Metabiologics Inc. (Madison, WI). The toxin was produced by a Hall A strain of *Clostridium botulinum*. Toxin activity for the holotoxin was 2.1 × 10^7^ MLD_50_/mg, according to the manufacturer. The human neuroblastoma cell line SH-SY5Y was obtained from the American Type Culture Collection (ATCC, Manassas, VA). Tissue culture media were ATCC-formulated Eagle’s Minimum Essential Medium (ATCC) with 10% fetal bovine serum (ATCC). Other materials and reagents include Ethanol (70% and 96–100%), Sterile, RNase-free pipet tips, 1.5 ml or 2 ml microcentrifuge tubes, Microcentrifuge for centrifugation at 4 °C and room temperature. 60 mm treated Petri dishes (Lab-Tek II, Nalge Nunc International, Naperville, IL). 4% Paraformaldehyde (Sigma-Aldrich, St. Louis, MO). miRNeasy Mini Kit (Qiagen).

### Cell culture and treatments

The SH-SY5Y cell line, derived from human brain neuroblastoma [45], grown and maintained as recommended by ATCC, was maintained with 10% FBS in 5% CO_2_/humidified air at 37 °C. SH-SY5Y cells grew as a mixture of floating and adherent cells. The base growth medium was 1:1 mixture of ATCC-formulated Eagle’s Minimum Essential Medium and F12 Medium. 10% of fetal bovine serum was added to the base growth medium to complete the growth medium. The SH-SY5Y cells were seeded at a density of 2 × 10^5^ cells/well in 60 mm treated Petri dishes and grew for 2 days when reaching the 80% confluence before treatment with serum-free media containing 5 nM of BoNT/A for 4 and 48-h in duplicates.

### RNA extraction

The following protocol was developed from manufacturer Qiagen’s manuals with modifications. At the end of treatment, QIAzol Lysis Reagent was used to added to disrupt and homogenize the SH-SY5Y cells. The upper aqueous phase was collected and treated with 100% ethanol and transferred into the RNeasy Mini column in 2 ml collection tube. After centrifugations, discarded the flow-through. RNeasy Mini columns were transferred to a new 1.5 ml collection tube. Pipetted 30 ml RNase-free water directly onto the RNeasy Mini column membrane. Closed lid, centrifuged for 1 min at 8000 Xg to elute. The concentration of RNA was determined by measuring the absorbance at 260 nm in a spectrophotometer.

### Microarray analysis

Genome analysis was carried out using Affymetrix U133 Plus 2 arrays. After preprocessing and normalization of the data, differentially expressed genes were identified by performing an analysis of variance using Partek Genomics Suite software (Partek Inc., St. Louis, MO). A gene list was created by filtering for *p*-value < 0.01. This subset of 1368 genes was further analyzed using the Database for Annotation, Visualization, and Integrated Discovery (DAVID) v6.8 to identify biologically enriched groups (Huang da, [19, 20]).

## Results

**Fig. 1:**
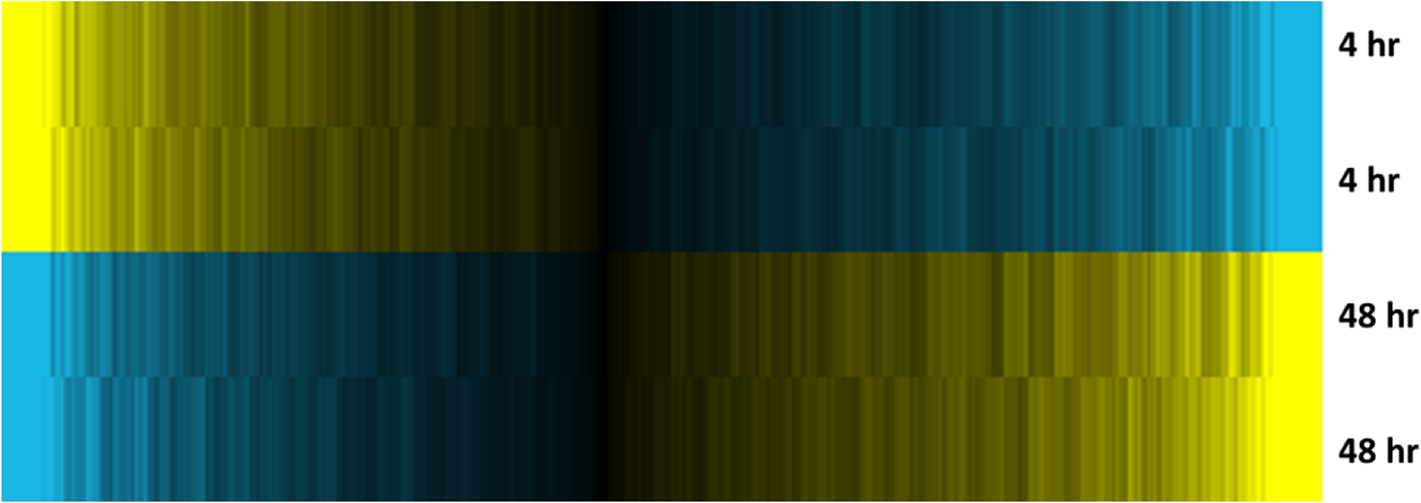
Heat map presentation of the differentially expressed genes after BoNT/A 4 and 48-h treatment. The heat map includes 1368 probes after running the ANOVA analysis with unadjusted *p*-value < 0.01. The values are mean centered with yellow indicating higher expression and blue indicating lower expression. Compared to the 4-h treatment, the 48-h treatment induces 744 up-regulated and 624 down-regulated genes

Our current study identified 1368 differentially expressed genes between the two time points. As presented in the heat map in Fig. 1, 744 genes were up-regulated, and 624 genes were down-regulated, indicating that there are significant differences between the gene expression profile for 48 and 4-h treatment of the SH-SY5Y cells.

**Fig. 2:**
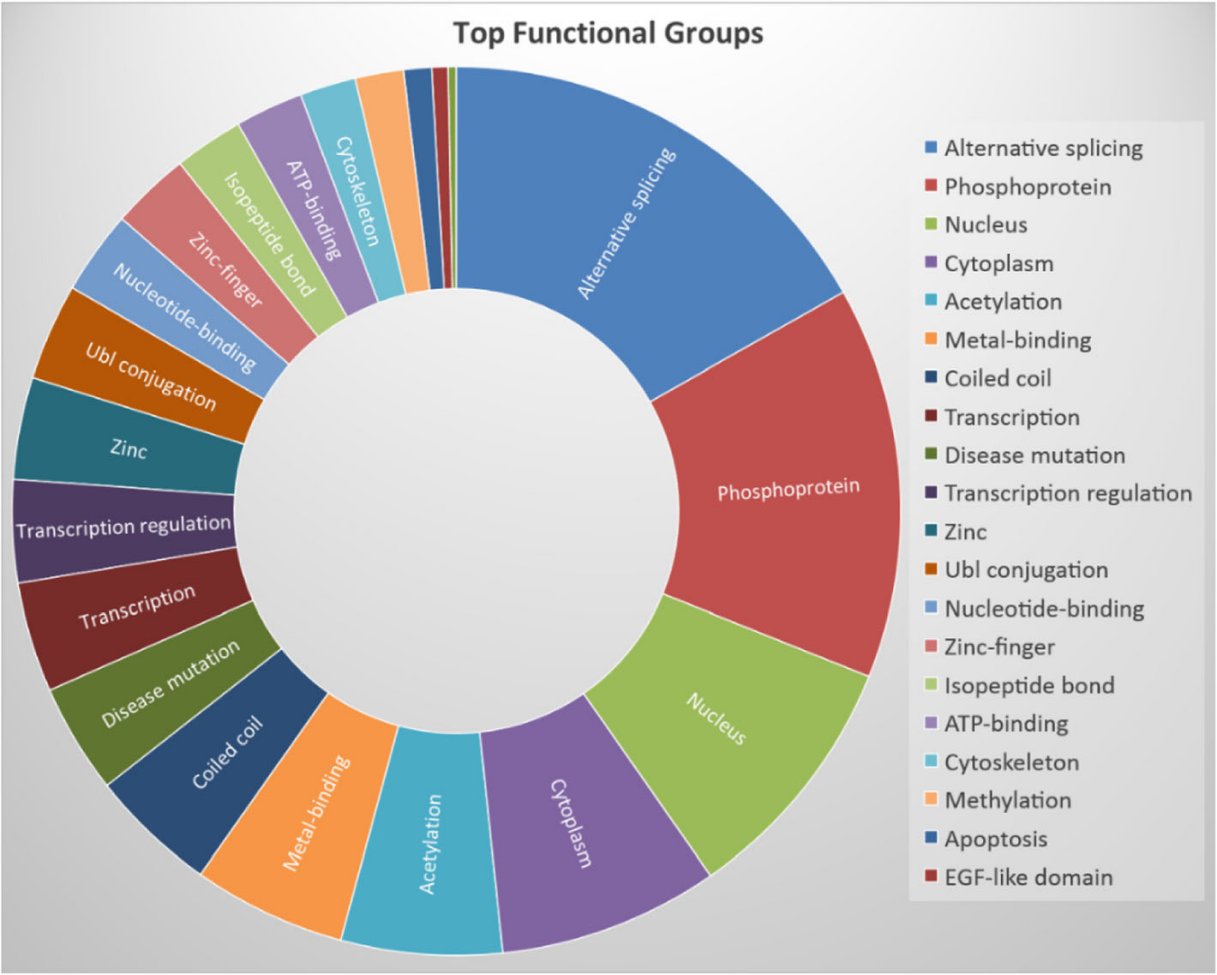
Proportions of numbers of genes involved in the top 20 Uniprot functional groups

Among the 1368 differentially expressed genes of the 48-h to 4-h treatment, we conducted DAVID analysis and identified 122 functional groups that have been significantly changed in the SH-SY5Y cells. Figure 2 shows the functional annotation chart of the top 20 functional groups according to the number of genes involved.

We took a closer look into the five groups that involved the most number of genes out of the 20 top functional groups and included the results in Table 1. The results table contains the numbers of involved genes, *p*-value, the numbers of up- or down-regulated genes (probe), and the fold change range for up- and down-regulation. These five groups are alternative splicing, phosphoprotein, nucleus, cytoplasm, and acetylation. These results indicate that following the entrance of BoNT/A into the SH-SY5Y cells after 4-h treatment, when the BoNT/A incubation reaches the 48-h, different cellular transcription, modification, and signaling transduction pathways ensue with profound cellular responses resulting from dramatic gene fold changes.

**Table 1:** Top five groups among the 122 identified DAVID functional groups

Gene Function Group	# of Genes count	% of Genes	***p***-Value	Probe # with > 2 fold change	Fold change range
Alternative splicing	660	60.44	2.74E-21	79↑ 64↓	19.41 ~ 2.03 26.63 ~ 2.02
Protein binding	566	51.83	5.45E-12	47↑ 66↓	32.28 ~ 2.03 31.23 ~ 2.01
Phosphoprotein	560	51.28	3.77E-25	54↑ 64↓	32.28 ~ 2.03 31.23 ~ 2.01
Splice variant	517	47.34	1.27E-18	66↑ 52↓	11.66 ~ 2.03 26.63 ~ 2.01
Nucleus	363	33.24	1.51E-14	29↑ 34↓	16.95 ~ 2.03 31.23 ~ 2.01

We have further investigated the major annotation groups that are involved in neuronal and biological functions. The complete gene list with more than 2-fold change are shown in the following supplement tables: neurological function (Supplement Table 1), neurological cellular response (Supplement Table 2), cell structure related function (Supplement Table 3), and the cell fate determination (Supplement Table 4). In each of these groups, genes were listed according to their DAVID annotation group with the fold change of up- or down-regulation.

Based on the results in Supplement Table 1 through 4, we generated Table 2 below that provides a highlight of the genes with more than 8-fold change after 48-h treatment. The genes are listed according to their annotation groups.

**Table 2:** BoNT/A effects with gene list and fold changes from the DAVID Analysis

DAVID Analysis: Functional Annotation Groups	Gene Title (Gene Symbol)	Fold change 48 h vs. 4 h
Nervous system development	cholinergic receptor, muscarinic 3 (CHRM3) sparc/osteonectin, cwcv and kazal-like domains proteoglycan (testican) 1 (SPOCK1) neural epidermal growth factor-like EGFL like 1 (NELL1)	32.28 ↑ 17.07 ↑ 16.95↑
Proteinaceous extracellular matrix	collagen, type I, alpha 2 (COL1A2)	11.99 ↓
Signaling pathways regulating pluripotency of stem cells	inhibitor of DNA binding 3, dominant negative helix-loop-helix protein (ID3) inhibitor of DNA binding 2, dominant negative helix-loop-helix protein (ID2)	8.13 ↓ 31.23 ↓
Cellular function and signal Transduction	Rho GTPase activating protein 36 (ARHGAP36)	11.66↑
Apoptosis	insulin like growth factor binding protein 3 (IGFBP3) serum/glucocorticoid regulated kinase 1 (SGK1)	13.26 ↓ 26.63 ↓

### Dramatic up-regulation of genes involved in acetylcholine receptor and other extracellular components

In the nervous system development annotation group, three genes were significantly up-regulated after 48-h treatment: CHRM3, SPOCK1, and NELL1.

The cholinergic receptor muscarinic 3 (CHRM3) that encodes the CHRM3 receptor has a 32.28-fold increase (*p* < 0.002). The muscarinic receptors (MRs) are G protein-coupled receptors that mediate cholinergic neurotransmission [16, 44]. The CHRM3 receptor responds to the transmitter acetylcholine [44]. The role of muscarinic receptors in the contraction of smooth muscle, particularly airway, ileum, iris, and bladder, is considered a classical muscarinic response mediated primarily by M3-muscarinic receptors expressed on the smooth muscle cells [9]. Five MR are identified (designated M1R–M5R, encoded by CHRM1–5). Post-MR signaling involves activation of phospholipase C with subsequent changes in cellular levels of inositol phosphate, cAMP, and calcium [34].

The sparc/osteonectin cwcv and kazal-like domains proteoglycan (testican) 1 (SPOCK1) gene that encodes the protein Testican has a 17.07-fold increase (*p* < 0.002). Testican is a human testicular proteoglycan which contains the extracellular calcium-binding module [3]. Immunostaining in the adult healthy mouse muscle has shown the co-localization of SPOCK and acetylcholine receptor (AChR) clusters [10].

The NELL1 gene, which is a secretory osteogenic protein and functions as an extracellular matrix component [31], has a 16.95 fold increase (*p* < 0.002). The dramatic expression increase of this group of genes after 48-h treatment indicates that after the acetylcholine release being blocked by BoNT/A, the genes involved in the expression of the acetylcholine responsive receptor, the extracellular components in the SH-SY5Y cells are dramatically up-regulated, highly possible to compensate the transmitter loss.

Another significantly up-regulated gene is Rho GTPase activating protein 36 (ARHGAP36), whose expression increased 11.66-fold (*p* < 0.003). Rho is present in organisms from yeast to mammals. This Ras homolog of small GTPase shuttles between the active GTP-bound form and the inactive GDP-bound form. The primary functions of Rho include a switch in stimulus-evoked cell adhesion and motility, enhancement of contractile responses, and cytokinesis [38]. In these actions, Rho directs the actin cytoskeleton’s reorganization at a specific time and a particular site in the cell [18]. ARHGAP36 is expressed in neuroblastoma cells and promotes aberrant activation of the Hedgehog pathway and inhibits Protein kinase A (PKA) signaling [15]. PKA is a key mediator of cAMP signaling downstream of G-protein-coupled receptors, a signaling pathway conserved in all eukaryotes. The up-regulation of ARHGAP36, in turn, dampens the sensitivity of cells to cAMP [15].

### Significant downregulation of genes for collagen deposition and cellular differentiation and apoptotic factors

Collagen type I makes up 90% of the collagen in the human body, and its function is to give the tissue resistance to force. Collagen type I is also one of the main components of the human skin dermis. The gene COL1A1 and COL1A2 encode the pro-alpha 1 and pro-alpha 2 chains of type I collagen, respectively [41]. Compared to 4-h treatment, we observed an 11.99-fold decrease (*p* < 0.009) of COL1A2 gene expression after 48 h. However, the Collagen-degrading matrix metalloproteinases (MMPs) gene expression did not significantly differentiate between our 48 and 4-h treatment. This finding indicates that although BoNT/A has down-regulation effects on the collagen extracellular matrix formation genes, the collagen degradation genes are not influenced.

We have also identified the downregulation of several genes that are involved in cell differentiation and survival. There are four known inhibitors of DNA binding (ID) proteins (ID1, ID2, ID3, andID4) which share a homologous HLH domain, but lack the basic DNA binding region. The basic helix-loop-helix (bHLH) family of transcription factors are critical cell type determinants that play essential roles in cellular differentiation [33]. We observed an 8.13 (*p* < 0.006) and 31.23 (*p* < 0.001)-fold down-regulation of ID3 and ID2, respectively. ID3 also regulates cytokines, including chemokine (C–X–C motif) ligand 1, previously known as GRO1, interleukin-6 (IL-6) and IL-8 [24]. We have observed a 13.26 fold decrease (*p* < 0.005) in the insulin-like growth factor binding protein 3 (IGFBP3). IGFBP regulates the bioavailability and bioactivity of insulin-like growth factor (IGF). The major IGFBP in serum is IGFBP-3. IGFBP-3 regulates IGF bioactivity and also independently modulates cell growth and survival (Dake, Boes, Bach, & Bar, 2004). There is also a 26.63-fold decrease (*p* < 0.007) of serum- and glucocorticoid-inducible kinase 1 (SGK1) genes that belong to the AGC family of kinases and have been shown to have various cellular functions, including the promotion of cell survival.

### BoNT/A evokes profound up and down-regulation of genes to impact physiological processes in SH-SY5Y cells

**Fig. 3:**
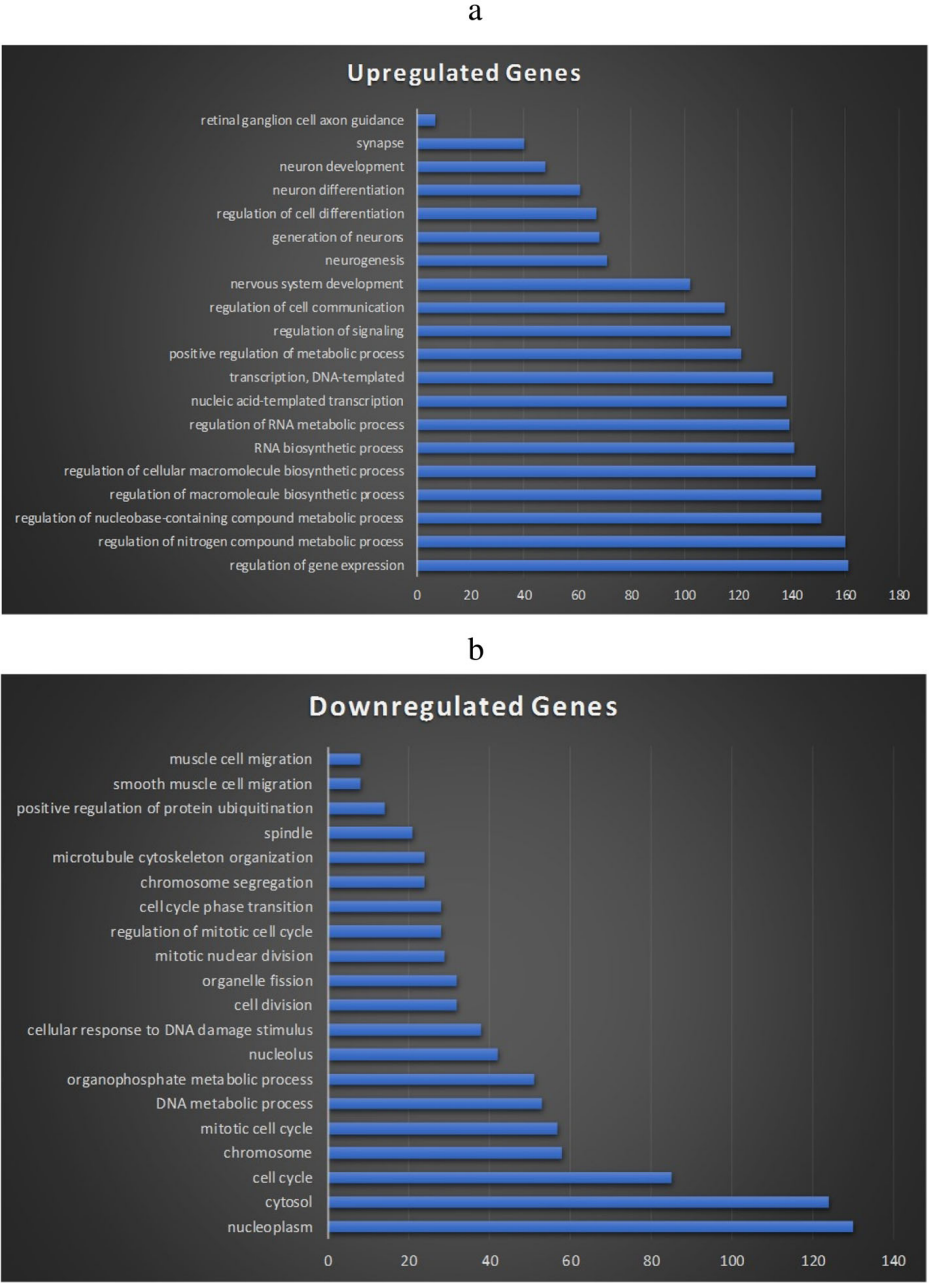
Functional groups listed by the up- or down-regulated set and the numbers of genes involved in each function group. **a**, 20 top functions for upregulated groups and **b**, 20 top functions for down-regulated groups

Besides looking into individual genes, to generate a thorough understanding of the effects of BoNT/A on the neurological and biological annotation groups, we ran a further analysis of the genes and determined whether the 48-h treatment evokes the up- or down-regulation of different annotation groups as shown in Fig. 3. Interestingly, there were no overlaps in the functional groups between the two up- and down-regulation group sets. We have also noticed that the up-regulated groups contained neuronal cell development, nervous system development, and metabolic processes; the down-regulated groups contained many chromosomes and cell cycle categories. These data indicate that BoNT/A treatment has profound effects on the neuronal and non-neuronal functions of SH-SY5Y cells. Moreover, the up and down-regulation of genes are coordinated to impact physiological processes.

## Discussion

Nerve terminal intoxication and the medical application of BoNT/A are fully reversible and are not reported to lead to neurodegeneration. After the synaptic blockade of cholinergic nerve terminals, new synapses are formed by the neuron (sprouting) to replace its original ones [13]. In the cellular model of cerebellar granule cells, BoNT/A did not trigger neurite degeneration or apoptosis up to 72-h treatment [5]. In this study, we have demonstrated that BoNT/A significantly modified genes involved in the annotation group of nervous system development and the proteinaceous extracellular matrix. The three genes CHRM3, SPOCK1, and NELL1 have been increased 32.28, 17.07, and 16.95 fold, respectively (Table 2). A study in the human induced pluripotent stem cell (hiPSC)-derived neurons found that BoNT/A induced the upregulation of CHRM3 by 9 fold compared to the non-treated control which promotes the neurite outgrowth [21, 48]. The SPOCK1 gene encoded the protein testican whose functions are to regulate cell shape and gene expression, cell adhesion processes, and neurogenesis [29]. This protein has also been shown to contribute to neuron-neuron synapse development and is involved in neuron-muscle synapse development [27, 35]. In a mature mouse model, the co-localization of SPOCK protein and acetylcholine receptor (AChR) clusters has been observed [10]. But botulinum toxins have been shown to induce a remodeling of AChR clusters [28]. AChR clusters devoid of SPOCK were observed after BoNT/A injection in an extensor digitorum longus muscle model [10]. In our study, this effect may also interact with the up-regulation of CHRM3, which activates the phospholipase C and, in turn, modifies the cellular level of cAMP. The evoking of a series of signal transduction effort in the cell after BoNT/A 48-h treatment demonstrates that the modification of cellular function and signal transduction are part of the on-site effects of BoNT/A. These data indicate that although we observed the significant up-regulation of CHRM3 and SPOCK1 genes, which may promote the increase of the expression of the receptors in the cells, this doesn’t guarantee the rise in the co-localization of the clusters of the related receptors. This finding calls attention to the further investigation of these receptors and their downstream signaling after BoNT/A treatment.

In addition to the genetic mechanism involved in the intoxication recovery, we have also identified the effects of BoNT/A in other biological functions as a neuromedicine. BoNT has been shown to inhibit hypertrophic scar formation [55] and modulate the release of various neurotransmitters, such as glutamate and substance P, which will help reduce scar hypertrophy [2]. We have identified the expression of different genes involved in collagen deposition in hypertrophic scars, and neurotransmitters involved in scar formation (Table 2 and Supplement Table 2). We observe an 11.99 fold decrease of the COL1A2 gene expression after 48-h treatment compared to 4-h. The gene COL1A1 and COL1A2 encode the pro-alpha 1 and pro-alpha 2 chains of type I collagen. Collagen type I makes up 90% of the collagen in the human body, and its function is to give the tissue resistance to force. Collagen type I is also one of the main components of the human skin dermis [41]. In our study, the Collagen-degrading matrix metalloproteinases (MMPs) gene expression did not significantly differentiate between our 48 and 4-h treatment groups. BoNT/A has been shown to block the syntheses of collagen types 1 and 3 in human fibroblasts upon the stimulation with TGF (S [25].). In another study on the human dermal fibroblasts, type III collagen mRNA expression decreased significantly when BoNT/A was administrated on KFs regardless of the presence or absence of TGF-ß [40].

It has been reported that BoNT/A inhibits the release of various neurotransmitters (e.g., glutamate and substance P), which inhibits the release of inflammatory mediators such as bradykinin, prostaglandins, and serotonin.6 [2]. A previous microarray study in SH-SY5Y cells from our group found that there were significant changes in genes involved in neuroinflammatory, ubiquitin-proteasome degradation, and calcium signaling after BoNT/A treatment compared to the untreated control culture [51]. Our data (Supplement Table 3) shows the profound effects of BoNT/A in the cytoskeleton involved genes that related to vesicle trafficking, axonal growth, and cell migration after 48-h treatment. Also, as shown in Table 2, the signaling pathways regulating the pluripotency of stem cells, cell division, and apoptosis functions have also been significantly modified by the 48-h treatment. Furthermore, the proteins that affect the phosphorylation pathways of cytoskeletal remodeling include the Rho GTPase, Ras-GAP SH3 binding protein, Rho GTPase activating protein, actin-related protein, and actin [30].. We have also observed a 26.6-fold decrease of SGK1 genes. Several observations point to a physiological role of SGK1 in cell cycle progression (Buse et al., 1999) and cell survival pathways. The upregulation of SGK1 strongly correlates with the occurrence of cell death (Schoenebeck et al., 2005). Our data indicate that BoNT/A has a strong inhibition effect on SGK1 expression, which may prevent the apoptosis of the SH-SY5Y cells.

None of the currently available publications have explored the effects of BoNT/A on the inhibition of DNA binding 2 or 3 (ID2 or ID3) expression. We have observed an 8.13 and 31.23 fold down-regulation of ID3 and ID2. In a study using human glioma cell line Hs683, candoxin, which is a three-finger neurotoxin purified from the venom of the Malayan krait (*Bungarus candidus*) snake has been shown to downregulate ID3 which inhibits glioma cell proliferation [23]. The cytokines that are regulated by ID3 genes, including chemokine (C–X–C motif) ligand one previously known as GRO1, interleukin-6 (IL-6), and IL-8 [24]. Our data indicate that BoNT/A can modify DNA binding factors, which may link to the longevity of BoNT/A treatment since the BoNT/A effect is extended into the transcriptional regulation and is not solely determined by the toxin protein half-life in the application sites. This observation sheds light on another mechanism to explain why the on-site effects of the BoNT/A treatment can last up to 16 weeks.

One of the advantages of BoNT/A medicinal application is the local effect at the neuromuscular junction (NMJ), resulting in flaccid paralysis. It has been believed that BoNT inhibits synaptic transmission only near the site of injection [53]. However, there has been evidence questioning this idea after physicians utilized this agent in human patients. For example, after peripheral injection of BoNT/A, the possibility that BoNT/A moves within networks of neurons to affect circuit function has been raised with the observation of reciprocal inhibition between agonist and antagonist muscles [4, 32]. Secondly, subsequent to the purely local effects on the initial uptake neurons, upstream of the injection site has shown the compensatory reorganization and remodeling of neuronal networks [1, 7, 12]. There has been another study conducted with BoNT/A and D in the compartmentalized microfluidic devices; the injection has undergone interneuronal transfer to affect networks of neurons [6]. These findings suggest a possible mechanism for the potential side effects after the BoNT application.

## Conclusion

The current study’s primary conclusion is that botulinum neurotoxin type A can cause dramatic neurological and biological effects on SH-SY5Y neuroblastoma cells. From the time BoNT/A enters the SH-SY5Y cells at 4-h to evoke on-site effects of BoNT/A after 48-h, the toxin has profound influences on gene expression profiles of SH-SY5Y cells. We identified significant expression changes of genes in the functional groups of nervous system development, proteinaceous extracellular matrix, signaling pathways regulating pluripotency of stem cells, cellular function and signal transduction, and apoptosis. Up-regulated groups contained neuronal cell development, nervous system development, and metabolic processes, while the down-regulated groups contained many chromosomes and cell cycle categories. Collectively, this experimental evidence strongly supports the overall conclusion that BoNT/A has profound neurological and biological effects on human neuroblastoma SH-SY5Y cells, which extend beyond the neurotransmitter release blockage. Our research warrants a more in-depth understanding of the mechanism of the toxin’s effects after the administration, which will help to understand the development of botulism disease and its longevity and explain the effectiveness of BoNT-related biomedicine. In future studies, we plan to include treatment times longer than 48 h, which will provide more comprehensive data for the understanding of the mechanism of the toxin’s effects after administration. Our data and the clinical evidence demonstrate that BoNT interacts with host systems in a more sophisticated manner than was initially envisioned, prompting the need for further investigations of the neurons and non-neuronal biology after the BoNT application.

## Supplementary information

**Additional file 1: Table S1.** BoNT/A effects on the neurological system related functional groups with gene list and fold changes from the DAVID Analysis. **Table S2.** BoNT related cellular biological functions. **Table S3.** Cell structure related functional groups and gene fold changes. **Table S4.** Cell fate determination functional groups and gene fold changes.

## Data Availability

All data generated or analyzed during this study are included in this published article and its supplementary information files.

## Abbreviations

BoNT: *Botulinum neurotoxin*
BoNT/A: *Botulinum neurotoxin* type A
HC: Heavy chain
LC: Light chain
IGF-1: Insulin-like growth factor-1
IL-6: Interleukin-6
