# Supplementary material for: Dramatic neurological and biological effects by botulinum neurotoxin type A on SH-SY5Y neuroblastoma cells, beyond the blockade of neurotransmitter release
Source: BMC Pharmacol Toxicol. 2020 Sep 5;21:66. doi: 10.1186/s40360-020-00443-0 (PMC7487822; doi:10.1186/s40360-020-00443-0)
Supplement: Supplementary file 1 — Additional file 1: Table S1. BoNT/A effects on the neurological system related functional groups with gene list and fold changes from the DAVID Analysis. Table S2. BoNT related cellular biological functions. Table S3. Cell structure related functional groups and gene fold changes. Table S4. Cell fate determination functional groups and gene fold changes. [file 40360_2020_443_MOESM1_ESM.docx]

**Supplementary tables:**

Table 1. BoNT/A effects on the neurological system related functional groups with gene list and fold changes from the DAVID Analysis

| DAVID Analysis: Functional Annotation Groups | Gene Title (Gene Symbol) | Fold change 48 hr vs 4 hr |
| --- | --- | --- |
| Nervous system development | cholinergic receptor, muscarinic 3 (CHRM3)  sparc/osteonectin, cwcv and kazal-like domains proteoglycan (testican) 1 (SPOCK1)  neural epidermal growth factor-like EGFL like 1 (NELL1)  Glial cell line-derived neurotrophic factor (GDNF) family receptor alpha 3 (GFRA3)  inhibin beta A (INHBA)  fibroblast growth factor 14 (FGF14)  chondroitin sulfate proteoglycan 5/neuroglycan C (CSPG5)  neurogranin (protein kinase C substrate, RC3) (NRGN)  ectodermal-neural cortex 1 (with BTB domain) (ENC1)  pleiotrophin (PTN) | 32.28 ↑  17.07 ↑  16.95 ↑  7.67 ↑  5.75 ↑  4.52 ↑  2.03 ↑  2.06 ↓  2.99 ↓  3.30 ↓ |
| Axon | potassium channel, voltage gated Shab related subfamily B, member 1 (KCNB1)  SRC kinase signaling inhibitor 1 (SRCIN1)  neurogranin (protein kinase C substrate, RC3) (NGRN)  neurofilament, light polypeptide (NEFL)  neurofilament, medium polypeptide (NEFM) | 4.18 ↑  2.32 ↑  2.06 ↓  2.71 ↓  5.84 ↓ |
| Cerebral cortex development | transforming, acidic coiled-coil containing protein 2 (TACC2)  phosphoribosylglycinamide formyltransferase, phosphoribosylglycinamide synthetase, phos (GART)  achaete-scute family bHLH transcription factor 1 (ASCL1)  neurofilament, light polypeptide (NEFL)  neurotrophic tyrosine kinase, receptor, type 2 (NTRK2) | 2.50 ↑  2.06 ↑  2.03 ↓  2.71 ↓  7.49 ↓ |

Table 2. BoNT related cellular biological functions

| Biological functions | Genes | Fold change 48 hr vs 4 hr |
| --- | --- | --- |
| Calcium channel involved in neuron regeneration and sprouting | potassium channel, calcium activated large conductance subfamily M alpha, member 1 (KCNMA1)  calcium channel, voltage-dependent, N type, alpha 1B subunit (CACNA1B)  RAS guanyl releasing protein 2 (calcium and DAG-regulated) (RASGRP2)  secretagogin, EF-hand calcium binding protein (SCGN) | 2.32 ↑  2.24 ↑  2.07 ↑  4.00 ↓ |
| Collagen deposition in hypertrophic scars | collagen, type I, alpha 2  fibroblast growth factor 14 (FGF14)  fibronectin leucine rich transmembrane protein 1 (FLRT1)  TIMP metallopeptidase inhibitor 3 (TIMP3)  sparc/osteonectin, cwcv and kazal-like domains proteoglycan (testican) 1 (SPOCK1) | 11.99 ↓  4.52 ↑  3.31 ↑  3.38 ↑  17.07 ↑ |
| Scar formation related neurotransmitters | glutamate receptor, ionotropic, AMPA 2 (GRIA2)  glutamate receptor interacting protein 1 (GRIP1)  solute carrier family 1 (glutamate/neutral amino acid transporter), member 4 (SLC1A4)  glutamate receptor, ionotropic, AMPA 3 (GRIA3)  5-hydroxytryptamine (serotonin) receptor 1E, G protein-coupled (HTR1E) | 2.11 ↑  2.08 ↑  2.46 ↓  5.73 ↓  2.11 ↑ |

Table 3. Cell structure related functional groups and gene fold changes

| DAVID Analysis: Functional Annotation Groups | Gene Title (Gene Symbol) | Fold change 48 hr vs 4 hr |
| --- | --- | --- |
| Proteinaceous extracellular matrix | sparc/osteonectin, cwcv and kazal-like domains proteoglycan (testican) 1 (SPOCK1)  TIMP metallopeptidase inhibitor 3 (TIMP3)  fibronectin leucine rich transmembrane protein 1 (FLRT1)  slit guidance ligand 1 (SLIT1)  slit guidance ligand 2(SLIT2)  cartilage acidic protein 1 collagen, type I, alpha 2 (CRTAC1)  Versican (VCAN)  ADAM metallopeptidase with thrombospondin type 1 motif 19 (ADAMTS19)  ADAM metallopeptidase with thrombospondin type 1 motif 17 (ADAMTS17)  collagen, type I, alpha 2 (COL1A2) | 17.07 ↑  3.38 ↑  3.31 ↑  2.54 ↑  2.46 ↑  2.24 ↓  2.38 ↓  2.64 ↓  3.05 ↓  11.99 ↓ |
| Cytoskeleton | cyclin-dependent kinase 6 (CDK6)  TRAF2 and NCK interacting kinase (TNIK)  transforming, acidic coiled-coil containing protein 2 (TACC2)  SRC kinase signaling inhibitor 1 (SRCIN1)  DENN/MADD domain containing 2A (DENND2A)  sperm associated antigen 5 (SPAG5)  POC1 centriolar protein A (POC1A)  checkpoint kinase 1 (CHEK1)  spindle and kinetochore associated complex subunit 2 (SKA2)  cell division cycle associated 8 (CDCA8)  tropomyosin 1 (alpha) (TPM1)  ectodermal-neural cortex 1 (with BTB domain) (ENC1)  neural precursor cell expressed, developmentally down-regulated 9 (NEDD9) | 3.08 ↑  2.77 ↑  2.50 ↑  2.32 ↑  2.13 ↑  2.07 ↓  2.11 ↓  2.12 ↓  2.29 ↓  2.30 ↓  2.44 ↓  2.99 ↓  5.30 ↓ |

Table 4. Cell fate determination functional groups and gene fold changes

| DAVID Analysis: Functional Annotation Groups | Gene Title (Gene Symbol) | Fold change 48 hr vs 4 hr |
| --- | --- | --- |
| Signaling pathways regulating pluripotency of stem cells | inhibin beta A (INHBA)  phosphatidylinositol-4,5-bisphosphate 3-kinase, catalytic subunit delta (PIK3CD)  bone morphogenetic protein receptor type IB (BMPR1B)  phosphoinositide-3-kinase, regulatory subunit 3 (gamma) (PIK3R3)  T-box 3 (TBX3)  inhibitor of DNA binding 3, dominant negative helix-loop-helix protein (ID3)  inhibitor of DNA binding 2, dominant negative helix-loop-helix protein (ID2) | 5.75 ↑  4.09 ↑  3.18 ↑  2.09 ↓  3.22 ↓  8.13 ↓  31.23 ↓ |
| Cell division | cyclin-dependent kinase 6 (CDK6)  sperm associated antigen 5 (SPAG5)  endosulfine alpha (ENSA)  spindle and kinetochore associated complex subunit 2 (SKA2)  cell division cycle associated 8 (CDCA8)  polyamine-modulated factor 1 (PMF1)  neural precursor cell expressed, developmentally down-regulated 9 (NEDD9) | 3.08 ↑  2.07 ↓  2.21 ↓  2.29 ↓  2.30 ↓  2.36 ↓  5.30 ↓ |
| Cellular function and signal transduction | Rho GTPase activating protein 36 (ARHGAP36)  RAB27B, member RAS oncogene family (RAB27B)  RAS guanyl releasing protein 2 (calcium and DAG-regulated) (RASGRP2)  RAB31, member RAS oncogene family (RAB31) | 11.66 ↑  6.26 ↑  2.07 ↑  2.56 ↓ |
| Apoptosis | pleckstrin homology-like domain, family A, member 1 (PHLDA1)  hypoxia inducible factor 3, alpha subunit (HIF3A)  serine/threonine kinase 4 (STK4)  protein kinase C, alpha (PRKCA)  paternally expressed 10 (PEG10)  caspase 6 (CASP6)  insulin like growth factor binding protein 3 (IGFBP3)  serum/glucocorticoid regulated kinase 1 (SGK1) | 3.51 ↑  2.45 ↑  2.23 ↑  2.01 ↓  2.15 ↓  2.15 ↓  13.26 ↓  26.63 ↓ |
